# Sialolipoma of the Parotid Gland: A Systematic Review

**DOI:** 10.7759/cureus.94803

**Published:** 2025-10-17

**Authors:** Theoklitos Tsaprazlis, Natalia Sinou, Georgios Kostakis, Amir Shihada, Dimitrios Filippou

**Affiliations:** 1 School of Dentistry, National and Kapodistrian University of Athens, Athens, GRC; 2 Department of Anatomy, National and Kapodistrian University of Athens School of Medicine, Athens, GRC; 3 Evgenidion Clinic Agia Trias S.A., National and Kapodistrian University of Athens, Athens, GRC

**Keywords:** lipomatous tumor, neoplasm, parotid gland, salivary gland, sialolipoma

## Abstract

Sialolipoma of the parotid gland is an uncommon benign neoplasm of the salivary glands, characterized histologically by the presence of mature adipose tissue intermixed with non-neoplastic salivary gland components, and sometimes featuring oncocytic or reactive alterations. The clinical, histopathological, and immunohistochemical characteristics of this condition, coupled with its similarity to various other parotid lesions in differential diagnosis, underline the necessity for accurate identification, with treatment typically involving parotidectomy, usually superficial, and aimed at preserving the facial nerve. A systematic electronic search was performed up to September 2025 across the PubMed and Scopus databases using pertinent Medical Subject Headings, without imposing any time limitations. A total of 86 studies were retrieved with the keywords “sialolipoma” AND “parotid”. Following the Preferred Reporting Items for Systematic Reviews and Meta-Analyses (PRISMA) guidelines, this systematic review includes 32 articles, which consist of 31 case reports and one retrospective descriptive study. The available evidence suggests an excellent prognosis, with recurrence being extremely rare, as only one recurrent case has been recorded. However, due to the small number of cases examined, the definitive recurrence rate is not well established, emphasizing the need for consistent follow-up to achieve more conclusive results.

## Introduction and background

Salivary gland tumors (SGTs) represent a small proportion of head and neck neoplasms, with the parotid gland being the most commonly affected major gland. Pleomorphic adenoma is the leading benign tumor, whereas lipomatous tumors in the parotid are incredibly rare. Notably, sialolipoma is a distinct histological variant characterized by the presence of mature adipose tissue intermixed with benign salivary gland structures like ducts and acini. Case reports have significantly contributed to the understanding of this unusual neoplasm by outlining its clinical presentations, imaging findings, and histopathological characteristics [1-4].

Epidemiological studies confirm the rarity of sialolipoma among SGTs, with a decade-long study revealing only a few occurrences [4]. Most patients are adults, though congenital and pediatric cases have been documented, including instances of recurrence in young children [5]. Some reports note atypical histological patterns [6] and larger tumors infiltrating adjacent areas, such as the parapharyngeal space [7]. The histological variability is underscored by the presence of oncocytic variations found in several studies [8], while other cases involving infants highlight the potential for early formation [9]. Moreover, rare cases like parosteal osseous lipoma affecting the temporal bone, with associated parotid involvement, have been recorded [10].

Sialolipoma holds significant clinical importance due to its capacity to resemble other benign parotid tumors like pleomorphic adenoma and lipoadenoma, which exhibit similar clinical and imaging traits [1-3]. Typically, it manifests as a slowly enlarging, painless, and movable parotid mass, which can lead to diagnostic uncertainty during preoperative assessments [5,6]. Given that imaging results are often ambiguous, histopathological verification is crucial to avert unnecessary extensive surgical procedures [7,8]. Complete surgical removal, most frequently via superficial parotidectomy, is curative in the majority of instances, with recurrence being extremely uncommon, even in congenital and pediatric cases [5,9,10]. Acknowledging this lesion's benign nature and positive prognosis helps clinicians avoid excessive treatment and ensures suitable long-term monitoring [11,12].

The precise pathogenesis of sialolipoma is not well understood. Various studies have suggested that it may result from the fatty replacement of salivary gland tissue due to chronic inflammation, ductal obstruction, or age-related changes [6,7]. Others posit a hamartomatous or developmental basis, supported by its presence in infants and congenital scenarios, indicating abnormal differentiation of salivary and mesenchymal elements during embryological development [5,9,10]. Oncocytic metaplasia and degenerative alterations noted in certain variants further bolster the notion of a reactive process rather than a neoplastic one [3,8,13]. Overall, the prevailing evidence suggests a multifaceted origin integrating both reactive and developmental factors that contribute to its morphological diversity [9,14].

Distinguishing sialolipoma from other lipomatous or mixed salivary gland lesions is vital for accurate diagnosis and management. Its clinical and radiological resemblance to pleomorphic adenoma, lipoadenoma, and diffuse lipomatosis often leads to diagnostic hurdles [2,3,6,9]. Histologically, sialolipoma comprises mature adipose tissue combined with normal-appearing acini and ducts, showing no cytological atypia or mitotic activity [1,5,7]. These characteristics differ from pleomorphic adenoma, which includes epithelial and stromal components with myxoid or chondroid differentiation, and from lipomatosis, in which fat diffuses into glandular tissue without encapsulation [9,12]. Given that fine-needle aspiration cytology (FNAC) results are frequently inconclusive in documented cases [8,11], and imaging characteristics often lack specificity, a definitive diagnosis hinges on histopathological analysis following surgical excision [15]. 

Due to its low incidence and histological variety, comprehending sialolipoma and its unique features is essential for healthcare providers and pathologists alike. Imaging modalities like computed tomography (CT) and magnetic resonance imaging (MRI) are useful for preoperative assessments, but a definitive diagnosis hinges on histopathological evaluation. Complete surgical excision is the primary treatment method to reduce recurrence risk.

This systematic review aims to compile and critically assess the findings from the existing literature on this rare parotid gland tumor variant, focusing on its clinical, macroscopic, and histopathological attributes while also considering various differential diagnoses associated with this neoplasm.

## Review

Materials and methods

This systematic review was conducted in accordance with the Preferred Reporting Items for Systematic Reviews and Meta-Analyses (PRISMA) guidelines, incorporating both a flow diagram and a checklist (see Appendices) to ensure a structured and transparent approach for conducting systematic reviews. To support our research, a comprehensive electronic search was carried out in the PubMed and Scopus databases using the keywords "sialolipoma" AND "parotid", without any restrictions. Given the significant number of lipomatous cases reported in the head and neck area, the authors concentrated exclusively on pertinent articles that documented diagnosed instances of sialolipoma within the parotid gland. The criteria for exclusion from this systematic review included studies not pertaining to the topic, articles focused on animal cases, non-availability of full-text, and any duplicate works. All authors contributed to the selection of studies, data extraction, and assessment of the review’s quality.

Results

**Figure 1:**
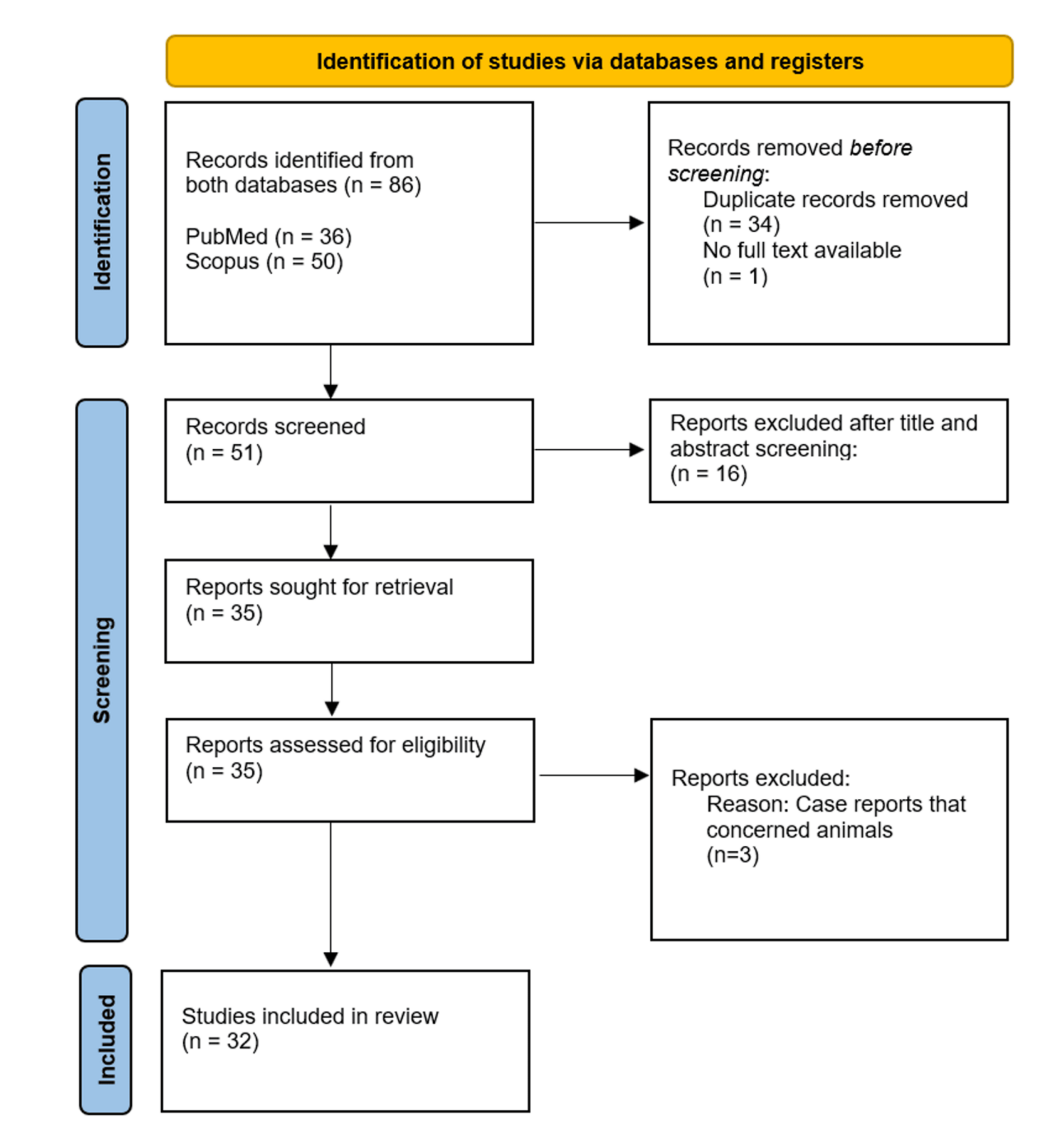
PRISMA flow diagram of the included studies. PRISMA, Preferred Reporting Items for Systematic Reviews.

The preliminary search returned 86 articles from the PubMed and Scopus databases, with no year of publication restrictions applied to the included studies. A total of 54 articles were excluded based on defined criteria; among them, 16 were unrelated to the systematic review’s topic, three concerned animal reports, one did not provide access to full text, while the remaining 34 were duplicates. A secondary manual literature search of the selected articles yielded no additional entries. Consequently, the overall number of the analyzed records utilized in this systematic review was 32, of which 31 articles were case reports and one was a retrospective descriptive study. The search approach for the specified databases is illustrated through the PRISMA flow diagram in Figure 1.

Discussion

Demographic Characteristics

The analytical systematic review of 32 articles included an assessment of 41 reported instances of sialolipoma located in the parotid gland [1-32]. Among these patients, 20 were female [1,2,6,8,11-13,15,16,18,23,25-28,32], 18 were male [3,5,7,9,10,14,17-22,24,26,29,31], and in three cases, there was no information provided on the patients’ gender [4,30]. Most of the patients were adults, comprising 28 individuals [1,3,6,8,10,11,13-18,20,21,24,26-29,31], while 11 were minors [2,5,7,9,12,19,22,23,25,30,32]. Ιn two cases, no exact data was provided regarding the age of the patients [4]. Their ages ranged from six weeks to 74 years, with an average age of 37.77 years [1-3,5-31].

Medical History

In terms of the medical history of patients diagnosed with sialolipoma in the parotid gland, the majority exhibited no signs of immunodeficiency [1,3,6,9,11,13,14,17,27,28,32] or associated illnesses [16,18,20,21,23,24,26,29,31]. Likewise, in pediatric cases, most reported full-term deliveries, uncomplicated pregnancies [19,22,25,32], and typical growth and development patterns [22,32]. Among the health issues noted in the medical histories of both adult and child patients were hypertension [1,14,17], epilepsy [8], type II diabetes [14,17], hyperlipidemia [14], prostate cancer [17], hypercholesterolemia [17], and back pain [17]. Furthermore, three patients showed similar lesions in the same area [2,5,18], and one case involved a patient with a family history of congenital defects [25].

Clinical and Radiological Findings 

Clinical observations indicate that sialolipoma can present as a mass, swelling, or lump in various regions, including the parotid [1,3,5-17,19,20,22,24,26-29,31], preauricular [3,6,23-25,32], angle of the mandible [6,21], submandibular [30], cervical [5], or neck [8] areas. The majority of patients experienced painless symptoms [2,3,11,14-16,21,23-25,29,31], with only three reporting pain [10,13,15]. During palpation, the tumors were noted to be soft [1,2,5,6,9,11,12,14,16,17,19,22-24,27-30,32], mobile [2,3,5,6,11-17,19,21,23-25,27,28,30,32], non-tender [1,3,5,6,11,15,17,19,22,23,25,27,28,30], well-defined [11,13,21,23,28], non-fluctuant [1], nodular [8], with irregular margins [22], tender [13], smooth [13], elastic [15], round [15], or oval [13]. In one instance, the mass's temperature during palpation was recorded as normal [6]. Tumor sizes varied between 1.5 cm and 10 cm in diameter [1-3,5-8,10-13,15-19,21,22,25-30,32], with some showing gradual growth over time [3,9-11,13-15,19,21,22,25,26,28-31]. Most patients did not exhibit any clinical involvement of the facial nerve [1,7,11-14,19,21,23-26,28,31,32], though one case did report paresthesia [2]. Additionally, cervical lymphadenopathy was mostly absent in the patients [3,11-15,19,20,23,25,28-30], with one exception [5]. The overlying skin appeared normal in five patients [14,16,21,30,32].

To ensure comprehensive evaluation and gather extensive information, various imaging techniques were utilized, including CT, MRI, ultrasonography, FNAC, and needle core biopsy [1,3,5-8,10-17,19-22,24-32]. For instance, CT imaging was performed on 17 patients [5,6,10,11,13-15,17,21,22,24,26-28,31], revealing a subcutaneous [5], encapsulated [17,27] mass characterized as hypodense [6,10,11,13,17,24,26], either homogeneous [27] or heterogeneous [13,17], well-demarcated [5,11,15,27,28,31], well-formed [21], and macro-lobulated [11], presenting fatty density [5,16,28] and non-enhancing septa [5]. After contrast administration [5,6,11,13,15,17,27,28], the lesion exhibited varying degrees of enhancement [15,17,27], while five patients showed no notable enhancement [5,6,11,13,28]. The mass was in close proximity [5] or projection [10] with the parotid gland [5,15,17,21,22,24,26-28], showing a capsule [10] and minor calcifications [10], impacting the deep cervical fascia beneath [28].

In one case, the lesion extended toward the interior of the mandibular ramus [10], with solitary lymph nodes detected along major neck vessels [10]. Some instances indicated no adjacent soft tissue involvement [6,28] or bony erosion [6], while a subtle stranding of overlying fat and skin was noted in one case [27]. The CT findings pertained to lipomatosis [13], benign tumor identification [14,31], and true lipoma [22]. MRI was conducted on nine patients [7,8,12,15,19,20,25,26,32], displaying a mass/lesion described morphologically as ovoid [15], lobulated [8], exophytic [8], or anteriorly located [20], exhibiting well-defined [25,32] and smoothly marginated [20] edges, typically regarded as benign [20] with occasional prominence [20]. Signal characteristics included hyperintensity [7,26] or isointensity to fat [8,32], at times showing scattered low-intensity regions [26].

The internal composition was characterized as solid [19], cystic [15,20], or of fatty nature [20]. Post-contrast administration, lesions displayed either contrast enhancement [7,20] or heterogeneous enhancement [19,32], with one lesion showcasing a pattern resembling a hemangioma [7]. The masses extended from the parotid gland [8,12,15,19,25], occupying parapharyngeal space [7], and sometimes extending to subcutaneous fat [15,25], masseter muscle [20], or subcutaneous tissue [20]. One case indicated unclear boundaries and signal intensity differences from the normal parotid gland [12], while another reported a lobulated isointense component [8]. For a recurrence case, MRI showed the emergence of a postoperative sialocele within the parotid gland [15].

Ultrasonography was performed on 16 cases [1,3,8,11-13,16,19,20,22,25,27,29,30,32], revealing a diffusely mature adipose [19] lesion/nodule characterized as solid [16,32], soft [12,13,25], and hypoechoic [11,16,29,30], with heterogeneous [13,22,25] internal features. Margins were reported as well-defined [11,29], well-encapsulated [21], and occasionally lobulated [29]. Some lesions appeared polycystic [20], while others lacked cystic areas [12]. Rarely, lesions were described as unclear [20]. The mass contained normal salivary gland acini [27] intermixed with occasional clusters of ductal epithelial cells [27] within a lipoidal, inflammatory backdrop [27], leading to considerations of an adenomatous mass [1], pleomorphic adenoma [3], or sialolipoma [19]. Additionally, intralesional vascularity was observed [11,13,25], resembling echo patterns of the left submandibular gland [25]. Slightly enlarged lymph nodes in the neck were noted in one instance [25].

FNAC was utilized in 13 cases [1,6,13,16,17,21,24,25,27,28,30-32], while a needle core biopsy was conducted on one patient [8], revealing mature adipocytes [24,30,31], cohesive clusters of small basaloid cells with minimal cytoplasm, a fine stippled chromatin pattern with occasional small nucleoli [25], microacinar growth intermixed with myoepithelial cells [25], salivary gland acini [27,30], and ducts [30] in a murky lipoidal inflammatory background [27], with negative results for malignancy [24]. This suggested various possibilities, including a cystic [1] or lipomatous [32] lesion, benign parotid tumor [6,31], sialoblastoma [25], or true lipoma [30].

Gross Pathology

The tumors' macroscopic appearance was documented in 27 patients [1-3,6-11,15-25,27-32]. They were often characterized as well circumscribed [1,8,15,17,22,32], encapsulated [20,21,23,27,29,30], and well defined [15,16,29]. Morphologically, the tumors presented as lobulated [9,11,16,18,19,22,25,27,32], with occasional irregularities [9,22,27], nodular [8,9,27], or globular [22], typically having a smooth external surface [6,25,30]. When cut, they were predominantly described as yellow [2,3,8,9,16,18-23,27,28,30,31], but at times tan-brown [3,8,11,15,16,19,27,32], and more rarely pale [1,17,25,28], greyish [2,22], white [23], or pinkish [6]. In terms of texture, the tumors were generally soft [1,2,15-17,20,28,32], fatty [8,10,15,18,24,30], or firm [1,17], with less frequent descriptions noting solid [9,27], homogeneous [20], granular [2], or well-preserved [2] characteristics. Notable features included masses intertwined with fibrous regions [23,25,30,31] or surrounded by small cysts [20]. Rare instances showed the cut surface with numerous empty spaces [6] or a surface resembling a lipoma [7]. In one unique case of parosteal osseous lipoma, the tumor was found to connect with a piece of bone tissue that was similar to a sessile exostosis [10], where three brownish areas of bony density were also noted [10]. The tumors varied in size, measuring between 1.1 × 0.6 × 0.4 cm and 9 × 8 × 4 cm [1-3,6-9,11,15-17,19,20,23-25,27-31], while two case reports recorded the masses at weights of 29.4 and 31 grams, respectively [19,20].

Histological Image

Histological analysis is a crucial step in diagnosing sialolipoma, having been conducted on 39 patients in the literature reviewed [1-3,5-32]. Regarding the tumor architecture, the lesions were generally well-circumscribed [1,3,16,17,25,26,30] and often encapsulated [3,5,6,8,10,11,14,15,19-21,23-27,30]; four cases were noted as serous lesions [18]. The predominant composition of these tumors was mature adipose tissue [1-3,5-32], commonly interspersed with fibrovascular septa [5,6,8,13,20,24,27,31], while rare instances lacked septa [2]. Additionally, adipose components were predominantly associated with glandular acini [1-3,5-31], ductal structures [1,3,6-10,12,14-16,19-31], lobules [2,3,5,8,10,14-16,19-26,29-31], and excretory ducts [5,8,10,14-16,19-26,29,31]. Small clusters of cells [13-15,17] and nerve bundles [11,24,26] were occasionally observed, often alongside chronic inflammatory infiltrates [1,12,18,20,25,30].

In terms of cellular characteristics, oncocytic differentiation was noted in five instances [3,8,13,17,26]. The epithelial cells exhibited a range of shapes [2], from cuboidal [6,30] to round [3,8] or oval [3]. Their nuclei were described as uniform [8], featuring granular chromatin [8], prominent nucleoli [3,8], and eosinophilic granular cytoplasm [3,8], lacking tonofilaments [26] or myofilaments [26]. Additional histological observations included frequent periductal lymphocytic infiltration [3,7-10,20,22,23,26,27], while focal sebaceous [3,10,18,20,23,26,30] and squamous [23,26] differentiation were also noted. Conversely, some reports indicated a lack of sebaceous differentiation in the ductal epithelium [3,18]. Moreover, degenerative and reactive changes like atrophy [8,10,12,14,20,23,25,29], dystrophy [10], fibrosis [8-10,12,18,20,23,25,29,30], vascular proliferation [9,10,18,20], myxomatous degeneration [10], fatty degeneration [24], and lymphoid hyperplasia [27] were documented.

In the unusual case of osseous lipoma, features such as immature cartilage, enchondral ossification, calcification, and bone trabeculae were identified [10]. As for negative findings, there was no evidence of cellular pleomorphism [3], atypia [3,6], mitotic activity [3], or malignant change [6,19,20,30]. Likewise, no signs of lymphovascular [3], perineural [3], or extracapsular [3] invasion were recorded, and the overlying epithelium was consistently reported as normal in one instance [2]. Lastly, regarding the quantitative distribution of the adipose component, it varied significantly among cases, ranging from 33% to over 90% of the tumor volume [3,8,10,11,13,16-20,26,30], which highlights the heterogeneity of these tumors.

Immunohistology 

An immunohistochemical analysis was performed on six patients [20,26], revealing that acinar cells exhibited expression of cytokeratin (AE1/AE3, CAM5.2), epithelial membrane antigen, secretory component, and α-amylase, while duct cells expressed cytokeratin (34βE12) and cytokeratin 14 [26]. The myoepithelial cells surrounding the acini and ducts were identified by their expression of cytokeratin 14, muscle-specific actin, and α-smooth muscle actin, with cytokeratin 14 also found in the striated and excretory ducts [26]. In addition, both acinar and adipose cells showed S100 positivity, while oncocytic cells exhibited strong mitochondrial immunoreactivity [26]. These observations confirmed that the glandular components within the tumor preserved their normal cellular phenotypes and structural integrity [26]. Ultimately, carcinoembryonic antigen was negative [26], and Ki67 proliferation levels were low [20,26], slightly elevated in instances involving sebaceous metaplasia [20].

Diagnosis

Diagnosing sialolipoma can be challenging due to its varied histopathological presentations and the multitude of neoplasms and conditions it can resemble. For a thorough diagnosis, the techniques employed in each case predominantly included histopathologic examination [1-3,5-32], with additional support from CT [5,6,10,11,13-15,17,21,22,24,26-28,31], ultrasonography [1,3,8,11-13,16,19,20,22,25,29,30,32], MRI [7,8,12,15,19,20,25,26,32], and lastly FNAC [1,6,8,16,24,30-32]. In six instances, immunohistochemistry was also utilized in the diagnostic process [20,26]. However, FNAC yielded inconclusive results in five patients [13,21,25,27,28], along with a patient who had undergone two needle core biopsies [8].

Besides the typical sialolipoma [1,2,4,6,11,13-18,20,21,23,24,26-29,31,32], other identified variants included congenital sialolipoma [5,7,9,12,19,22,25,30], oncocytic sialolipoma [3,8], and ossifying sialolipoma [10]. It is essential for clinicians to be aware of all possible differential diagnoses associated with this condition. Furthermore, it is important to mention that Doğan et al. (2009) focused solely on benign parotid tumors [21], which excludes their classification from Table 1, which lists the neoplasms and conditions relevant to the differential diagnosis of sialolipoma.

**Table 1:** Neoplastic and non-neoplastic conditions included in the differential diagnosis for sialolipoma with the associated bibliographic references.

Neoplastic and Non-neoplastic Conditions	Bibliographic references
Pleiomorphic adenoma	[1-3,5,6,9,11,12,14,16,17,19,20,22-29,31]
Lipoadenoma	[1,2,5,6,8,9,12,15,17-20,22-24,26,28,29,31,32]
Lipomatosis	[1,3,5,6,9,12,15,17,19,22-24,26-32]
True lipoma	[9,11-16,18,20,24,26,28,31,32]
Hemangioma	[5,7,13,22,25,30,32]
Spindle cell lipoma	[3,12,15,28,31,32]
Fibrolipoma	[3,12,28,31,32]
Sialoblastoma	[5,7,22,25,30]
Cystic hygroma	[5,7,19,22,25]
Branchial cleft cyst	[5,7,19,22,25]
Parotitis	[5,12,19,25,32]
Hamartoma	[5,22,26]
Mucoepidermoid carcinoma	[5,14,25]
Warthin’s tumor	[11,13,14]
Acinic cell carcinoma	[5,25]
Benign mixed tumour of the parotid	[13]
Sebaceous carcinoma	[23]
Sebaceous lymphadenoma	[23]
Mucoepidemioid tumor	[23]
Adenoid cystic carcinoma	[23]
Lip adenoma	[27]
Nodular oncocytic hyperplasia	[8]
Liposarcoma	[28]
Lipomatous atrophy	[30]
Lipoblastoma	[32]

Therapy

A review of case studies regarding the management of sialolipoma in the parotid gland showed that parotidectomy was the most frequently performed surgical procedure [1-3,5-17,19-29,31,32]. Specifically, 27 patients had superficial parotidectomy [1,3,5,6,9-11,13-17,20-23,25-28,31,32], while seven underwent total parotidectomy [2,7,8,12,19,24,29] and one patient had deep parotid lobectomy [15]. These procedures included preservation and dissection of the facial nerve [1-3,5-17,19-29,31,32] [2,7,8,11,12,15,19,22,24,29,31].

Prognosis and Follow-up 

In the documented cases of patients undergoing treatment for sialolipoma in the parotid gland, 30 cases provided detailed information regarding prognosis and follow-up assessments [1,2,5,7,9,11-16,18-22,24-29,31,32]. The postoperative recovery was generally noted as uneventful [1,2,7,11,13-15,20,21,25,27,29,31,32], with preserved facial nerve function [1,2,7]. Nevertheless, some patients experienced complications such as peripheral facial nerve palsy [12,22,28] and facial nerve weakness [19,24], leading to challenges with eye closure and a slight droop of the mouth corner [22]. Follow-up durations ranged from one month to as long as seven years and seven months post treatment [1,2,5,7,9,11-16,18,19,22,24-29,31]. Most cases reported no recurrence [1,2,5,7,9,11-16,18,19,22,24-29,31,32], while a single recurrence was documented at the three-month follow-up [15].

## Conclusions

Sialolipoma of the parotid gland is a rare benign tumor of the salivary gland that can manifest in individuals of any age, most commonly in adults, though it is also seen in pediatric and congenital instances. Histopathologically, it features mature adipose tissue intermixed with non-neoplastic salivary components like acini and ducts, which may occasionally display oncocytic changes or other reactive characteristics. Imaging studies might indicate a benign lipomatous mass, but a conclusive diagnosis depends on histopathological examination, with immunohistochemistry providing additional support.

Clinically, the tumor typically appears as a swelling in the parotid area, and the main differential diagnoses include pleomorphic adenoma, lipoadenoma, and lipomatosis, highlighting the importance of thorough evaluation. Surgical intervention is the primary treatment approach, usually involving superficial parotidectomy while preserving the facial nerve, and the prognosis is excellent since recurrences are extremely rare. However, due to the limited number of cases recorded, precise recurrence rates cannot yet be determined, necessitating regular follow-ups for reliable long-term assessment.

## Appendices

**Table 2:** PRISMA 2020 checklist. PRISMA, Preferred Reporting Items for Systematic Reviews

Section and Topic	Item #	Checklist item	Location where item is reported
TITLE			
Title	1	Sialolipoma of the Parotid Gland: A Systematic Review	1^st^ page
ABSTRACT			
Abstract	2	Provides a structured summary including background, objectives, methods (PubMed and Scopus search until September 2025), results (32 included studies: 31 case reports and 1 retrospective study), and conclusions (rare, benign, extremely rare recurrences).	2^nd^ paragraph
INTRODUCTION			
Rationale	3	Explains the rarity of sialolipoma and the need to systematically compile clinical, imaging, and histopathological features.	1^st^ paragraph
Objectives	4	The article aims to compile and critically evaluate available evidence on parotid sialolipoma, focusing on clinical, macroscopic, histopathological characteristics, and differential diagnoses.	2^nd^ paragraph
METHODS			
Eligibility criteria	5	Included: articles reporting diagnosed parotid sialolipoma. Excluded: animal studies, unrelated topics, duplicates, and articles without full-text access.	1^st^ paragraph
Information sources	6	Research via PubMed and Scopus. The search was performed up to September 2025.	1^st^ paragraph
Search strategy	7	Keywords: “sialolipoma” AND “parotid”; no time restrictions; supplemented by manual search.	1^st^ paragraph
Selection process	8	All authors participated in study selection, data extraction, and quality assessment.	1^st^ paragraph
Data collection process	9	Data collected included demographics, clinical findings, imaging results, histopathology, immunohistochemistry, treatment, and follow-up.	1^st^ paragraph
Data items	10	Outcomes sought: age, sex, medical history, clinical presentation, tumor type and size, imaging characteristics, histology and immunohistochemistry, surgical intervention, complications, and prognosis.	1^st^ paragraph
Study risk of bias assessment	11	Not explicitly detailed; authors note that quality assessment was performed but methods are not specified.	No assessment of risk of bias
Effect measures	12	No meta-analysis was performed; results are presented qualitatively with detailed narrative and tables.	
Synthesis methods	13a	Eligibility for Synthesis: Only studies reporting diagnosed parotid sialolipoma in humans were considered eligible. Case reports and retrospective descriptive studies were included, while animal studies, unrelated topics, duplicates, and inaccessible full-texts were excluded.	
	13b	Data Preparation: Data were extracted on patient demographics, clinical presentation, imaging findings, histopathology, immunohistochemistry, surgical intervention, complications, and follow-up outcomes. Missing or unclear data were recorded and addressed narratively.	
	13c	Methods of Synthesis: Given the heterogeneity of study designs and outcomes, a qualitative (narrative) synthesis was performed. Data were summarized descriptively, highlighting common patterns in clinical presentation, imaging, histopathology, and treatment outcomes. Tabular summaries were used to organize study characteristics.	1^st^ paragraph
	13d	Handling of Heterogeneity: Heterogeneity in patient age, tumor size, histopathological variants, and imaging modalities was addressed by stratifying findings into subgroups (e.g., adult vs. pediatric cases, imaging modality, tumor variant). Meta-analysis was not possible due to insufficient quantitative data.	1^st^ paragraph
	13e	Sensitivity Analyses: Sensitivity analyses were not performed due to the case report nature of the included studies and lack of standardized quantitative outcomes.	Not required because they are case reports
	13f	Certainty Assessment: Formal certainty or confidence assessment (e.g., GRADE) was not applied. Limitations of the evidence, including small sample size, case-report design, and heterogeneity, were acknowledged in the discussion.	Not required because they are case reports
Reporting bias assessment	14	No formal assessment performed. Recognized limitations include potential publication bias (case reports), missing data in several studies, and caution urged in interpretation.	Not required because they are case reports
Certainty assessment	15	Not formally assessed; narrative discussion notes limitations due to small sample size and heterogeneity.	
RESULTS			
Study selection	16a	86 records identified from PubMed and Scopus; 32 included (31 case reports, 1 retrospective study); 54 excluded (16 unrelated, 3 animal, 1 no full text, 34 duplicates).	1^st^ paragraph
	16b	Full-text exclusions: 1 inaccessible, remaining excluded due to irrelevance or duplication.	1^st^ paragraph
Study characteristics	17	32 studies summarized: 41 patients; age 6 weeks–74 years (mean 37.77); 20 females, 18 males, 3 unknown; clinical presentation, tumor location, size (1.5–10 cm), imaging modality (CT, MRI, USG, FNAC), histology, immunohistochemistry, surgical procedure, follow-up.	1^st^ paragraph
Risk of bias in studies	18	Not formally assessed; quality review mentioned but without detailed scoring.	Not required
Results of individual studies	19	Each study summarized with: demographics, clinical features (painless swelling, mass location), imaging results (CT: hypodense, fatty; MRI: hyperintense or isointense to fat; USG: hypoechoic, solid), histopathology (mature adipose with salivary elements, occasional oncocytic changes), immunohistochemistry (CK, S100, α-amylase), surgical procedure (superficial/total parotidectomy), complications, and follow-up outcomes.	Not required
Results of syntheses	20a	Clinical: predominantly painless parotid swelling; Imaging: benign lipomatous lesion; Histology: mature adipose tissue with normal salivary structures; Surgery: superficial parotidectomy with facial nerve preservation; Prognosis: excellent, 1 recurrence.	
	20b	No meta-analysis; heterogeneity precludes quantitative pooling.	Not required
	20c	Narrative stratification: adult vs pediatric, congenital vs acquired, imaging modality, tumor variant; discussed differences in tumor size, histology, and follow-up.	Not required
	20d	Not performed due to case report design and absence of standardized quantitative outcomes.	
Reporting biases	21	No formal assessment; narrative notes potential publication bias (case reports more likely published if unusual or favorable), and incomplete reporting in some studies.	
Certainty of evidence	22	Not formally assessed (no GRADE); narrative notes low certainty due to small sample size, heterogeneity, and predominance of case reports.	
DISCUSSION			
Discussion	23a	The review confirms that sialolipoma of the parotid gland is a rare benign tumor with excellent prognosis. Clinical presentation is most often a painless swelling in the parotid region. Imaging modalities (CT, MRI, USG) generally suggest a lipomatous lesion, but definitive diagnosis relies on histopathology. Histologically, tumors consist of mature adipose tissue intermixed with non-neoplastic salivary structures, occasionally showing oncocytic or reactive changes. Surgical intervention, typically superficial parotidectomy with facial nerve preservation, is the treatment of choice. These findings are consistent with previous isolated case reports and small series.	1^st^-8^th^ paragraph
	23b	Evidence is limited by the small number of reported cases (41 patients), predominance of case reports, and heterogeneous reporting of clinical, imaging, and histopathological data. Some studies lacked complete demographic or follow-up information. The rarity of the condition limits the ability to determine precise recurrence rates and long-term outcomes.	
	23c	The systematic review did not include a formal risk of bias assessment or certainty assessment (e.g., GRADE). No quantitative synthesis or meta-analysis was feasible due to heterogeneity. Searches were limited to PubMed and Scopus, and although manual searches were performed, relevant reports may have been missed. Data extraction relied on available reports, which varied in detail and quality.	No limitations of the review
	23d	Practice: Clinicians and pathologists should recognize sialolipoma as a rare benign parotid tumor and differentiate it from other lipomatous or salivary gland lesions. Policy: No specific policy recommendations, but documentation of rare cases in registries could improve understanding. Future research: Larger multicenter studies or registries are needed to better estimate recurrence rates, characterize histological variants, and establish long-term outcomes. Additional research on imaging correlations and immunohistochemical profiles may aid in preoperative diagnosis and surgical planning.	No implications of the results
OTHER INFORMATION			
Registration and protocol	24a	Not reported. No registration (e.g., PROSPERO) was mentioned for this systematic review.	1^st^ page (correspondence address)
	24b	Not reported. No publicly available protocol was cited; a formal protocol appears not to have been prepared prior to the review.	No protocol prepared
	24c	Not applicable. Since no protocol was registered or published, there were no amendments to report.	No amendments
Support	25	Not reported.	No sources of financial
Competing interests	26	Not reported.	No competing interests
Availability of data, code and other materials	27	Not reported.	None
